# Clinicopathological characteristics of pancreatic acinar cell metaplasia associated with *Helicobacter pylori* infection

**DOI:** 10.1186/s12876-022-02338-2

**Published:** 2022-06-07

**Authors:** Takafumi Fuchino, Yasuhiro Wada, Masaaki Kodama, Ken-ichi Mukaisho, Kazuhiro Mizukami, Tadayoshi Okimoto, Ryoji Kushima, Kazunari Murakami

**Affiliations:** 1 Department of Gastroenterology, Faculty of Medicine, Oita University, 1-1 Idaigaoka, Hasama-machi, Yufu, Oita 879-5593 Japan; 2 Faculty of Welfare and Health Science, Oita University, Oita, Japan; 3 Education Center for Medicine and Nursing, Faculty of Medicine, Shiga University of Medical Science, Otsu, Shiga Japan; 4 Department of Pathology, Faculty of Medicine, Shiga University of Medical Science, Otsu, Shiga Japan

**Keywords:** Pancreatic acinar cell metaplasia, Gastric mucosa, *Helicobacter pylori*, Gastric cancer

## Abstract

**Background:**

Pancreatic acinar cell metaplasia (PACM) has been rarely reported in the gastric mucosa. In the present study, we aimed to elucidate the clinical and pathological characteristics of PACM associated with *Helicobacter pylori* (*H. pylori*).

**Method:**

5930 patients who underwent five- or two-point gastric biopsy according to the updated Sydney system (USS) by upper gastrointestinal endoscopy were enrolled. The patients were categorized into current *H. pylori* infection (CHI), post-*H. pylori* eradication (PHE), and non*-H. pylori* infection (NHI) groups according to the *H. pylori* infection status, and the frequency and location of PACM were compared. Additionally, a case–control study was performed to compare the USS scores between patients with CHI and PACM and those with CHI but not PACM.

**Result:**

The frequencies of PACM were 0.49% (10/2039), 0.75% (25/3332), and 0% (0/559) in the CHI, PHE, and NHI groups, respectively. PACM was found in the greater curvature of the antrum in 33 of the 35 patients with PACM. Among the patients with CHI, the inflammation scores in the greater curvature of the antrum and the greater curvature of the corpus were lower in patients with PACM than in those without PACM.

**Conclusion:**

Although rarely reported in the gastric mucosa, PACM was closely related to *H. pylori* infection, especially in the antrum, and was associated with relatively mild inflammation.

**Supplementary Information:**

The online version contains supplementary material available at 10.1186/s12876-022-02338-2.

## Background

Pancreatic acinar cell metaplasia (PACM), which is a rare finding in gastric mucosa, comprises clusters of pancreatic acinar cell-like cells that are mainly located in the gastric lamina propria [1]. PACM has been observed in the esophagogastric junction of patients with Barrett’s esophagus and in the greater curvature of the gastric corpus in patients with autoimmune gastritis [2, 3]. Unlike heterotopic pancreas, PACM lacks pancreatic ducts and islets of Langerhans [1, 4, 5]. Several studies have utilized immunohistochemical analysis to demonstrate that PACM is positive for lipase, trypsin, amylase, and chymotrypsin [1, 2, 4].

*Helicobacter pylori* (*H. pylori*) can cause gastric mucosal damage, which can present as gastric atrophy, intestinal metaplasia, pyloric metaplasia, or pseudopyloric metaplasia [6, 7]. Gastric atrophy and intestinal metaplasia caused by *H. pylori* infection can also occur as precancerous or paracancerous lesions in gastric cancer [6]; however, the association between PACM and *H. pylori* and the characteristics of PACM in the context of *H. pylori* have not been studied in detail.

Therefore, we investigated the clinicopathological characteristics of PACM in the gastric mucosa in a large cohort of over 6000 patients using histological and immunohistochemical analyses, with the aim to clarify the relationship between PACM and *H. pylori* and the effect of PACM on gastric mucosal damage.

## Methods

### Subjects

This was a retrospective, single-center cohort study including 6655 patients, including 3779 male and 2876 female patients, with a mean age of 61.0 ± 16.5 years, with available biopsy specimens collected during upper gastrointestinal endoscopy between January 1, 1998 and December 31, 2019 in Oita University Hospital. All clinical information for analyses, including personal medical history, endoscopy examination findings, and blood examination results, were obtained from the electronic medical records. This study was approved by the Research Ethics Committee of Oita University Hospital (approval number 2036).

### Evaluation of H. pylori status and PACM detection

Tissue culture, histological examination, rapid urease test (PyloriTek, Serim Research, Elkhart, IN, USA), and anti-pylori IgG-antibody (SRL, Tokyo, Japan) were used together for the diagnosis of *H. pylori* infection. *H. pylori* infection was defined as a serum anti-*H. pylori* antibody level ≥ 10 IU/L. Proton pump inhibitor (PPI)-based triple therapy was used for *H. pylori* eradication. Urea breath test (Otsuka, Tokushima, Japan) was performed four weeks after eradication treatment to confirm therapy success.

In the present study, patients with at least one positive diagnostic test for *H. pylori* infection were defined as those with current *H. pylori* infection (CHI). Patients with apparently recorded history of eradication treatment and negative results by all diagnostic tests were defined as those with post-*H. pylori* eradication (PHE). Patients who met the following criteria were defined as those without *H. pylori* infection (non-*H. pylori* infection; NHI): absence of anti-pylori IgG antibodies, negative findings of *H. pylori* infection by histological examination, absence of histological gastritis, and absence of gastric corpus atrophy by endoscopy [8]. The association between PACM and *H. pylori* was examined in patients categorized into the CHI, PHE, and NHI groups.

### Histological examination

We have taken biopsy specimens from five or two sites designated in the updated Sydney system (USS) (five- or two-point biopsy) for research of *H. pylori* gastritis for over 20 years [9]. Five-point biopsy specimens were collected from the greater curvature of the antrum (A2), the lesser curvature of the antrum (A1), the lesser curvature of the angulus (IA), the lesser curvature of the corpus (B1) and the greater curvature of the corpus (B2). Two-point biopsy specimens were collected from A2 and B2. A1 and A2 were located 2–3 cm superior to the pyloric ring, B1 was located approximately 4 cm proximal to the gastric angulus, and B2 was located 8 cm inferior to the cardia [9]. Based on USS, we took biopsy specimens from sites without abnormity as ulcers, erosions and cancer during the observation of esophagogastroduodenoscopy. The biopsy specimens were immediately placed in separate containers with 10% neutralized buffered formalin for fixation, followed by embedding in paraffin wax. The specimens were subsequently sliced into 3-μm-thick sections and stained with hematoxylin and eosin. In the present study, all of the biopsy specimens were actually reviewed using a microscope, not electronic archive, to identify PACM.

### Immunohistochemical staining

All specimens in which pancreatic acinar-like cells had been morphologically identified in hematoxylin/eosin-stained specimens were immunocytochemically stained. Immunohistochemical staining was executed with the ENVISION system (DAKO, Glostrup, Denmark) with antibodies against Bcl-10, α-amylase, MUC6, Ki-67, and pepsinogen-I. Anti-Bcl-10 was used as a sensitive and specific marker of pancreatic acinar cells and pancreatic metaplasia [10]. Following deparaffinization with xylene and washes with ethanol, endogenous peroxidase activity was blocked using 3% hydrogen peroxide (Wako, Osaka, Japan). The following primary antibodies were used after antigen retrieval in buffer at pH 9: mouse monoclonal antibody against Bcl-10 (1:100; Santa Cruz Biotechnology, Dallas, TX, USA), rabbit monoclonal antibody against α-amylase (1:100; Proteintech, Rosemont, IL, USA), mouse monoclonal antibody against Ki-67 (diluted; DAKO), and mouse monoclonal antibody against MUC6 (diluted; Leica Biosystems, Newcastle Upon Tyne, UK). The following primary antibody was used after antigen retrieval at pH6: mouse monoclonal antibody against pepsinogen-I (1:200; Bio-Rad, Hercules, CA, USA). A peroxidase-labeled goat anti-mouse secondary antibody, an ENVISION reagent (DAKO), was used to detect the anti-Bcl-10, anti-Ki-67, anti-MUC6, and anti-pepsinogen-I antibodies. A peroxidase-labeled goat anti-rabbit secondary antibody, an ENVISION reagent (DAKO), was used to detect the anti-α-amylase antibody. The slides were subsequently incubated with 3,3’-diaminobenzidine for 5 min, and hematoxylin staining was followed by dehydration, clearance, and mounting.

### Double immunofluorescence staining

Double immunofluorescence staining for Bcl-10 and MUC6 was performed to clarify the association between PACM and pyloric-type glands. Briefly, after deparaffinization and ethanol washes, the endogenous peroxidase was deactivated using 3% hydrogen peroxide solution and antigen retrieval was performed at pH 6. The following primary antibodies were used: mouse monoclonal antibody against Bcl-10 (1:100; Santa Cruz Biotechnology) and mouse monoclonal antibody against MUC6 antibody (1:4000; Abcam, Cambridge, England). An Alexa Flour 594-labeled wheat anti-IgG (1:200; Invitrogen, Carlsbad, MA, USA) was used as the secondary antibody against anti-Bcl-10, and an Alexa Flour 488-labeled wheat anti-IgG (1:200; Invitrogen) was used as the secondary antibody against anti-MUC6. DAPI (1:1000: Wako) was used for nuclear staining. All stained sections were examined using a KEYENCE BZ-9000 (Keyence, Osaka, Japan) all-in-one fluorescence light microscopy.

### Case–control study of PACM with CHI

A case–control study was performed to examine background factors associated with PACM in patients with CHI. Briefly, patients with CHI were categorized into those with PACM (PACM group) and those without PACM (non-PACM group) who were age- and sex-matched. Differences in USS scores for inflammation, activity, atrophy, and intestinal metaplasia were examined at two sites, A2 and B2, between the two groups. USS were scored as follows: 0, normal; 1, mild; 2, moderate; and 3, marked. Additionally, the degree of endoscopic atrophy according to the Kimura–Takemoto classification was compared between the two groups [11]. Briefly, endoscopic atrophic border was classified into 7 grades: C-0, no atrophy; C-1, closed border in the antrum; C-2, closed border in the distal corpus; C-3, closed border in the proximal corpus; O-1, open border in the lessor curvature; O-2, open border between lessor and greater curvatures; and O-3, open border in the greater curvature or pan-atrophy in the corpus without atrophic border. The following scores were used to determine the degree of endoscopic atrophy: 0, C-0; 1, C-1; 2, C-2; 3, C-3; 4, O-1; 5, O-2; and 6, O-3 [11]. Finally, serum gastrin level (RIA, SRL, Tokyo, Japan) in samples obtained immediately before upper gastrointestinal endoscopy was compared between the two groups in the case–control study.

### Statistical analysis

In the case–control study, the USS scores, atrophy scores and serum gastrin levels were presented as means with standard error of the mean. Differences in USS scores and atrophy scores between the groups were compared using the Mann–Whitney *U* test. Differences in serum gastrin levels between the groups were analyzed using Student’s *t* test. SPSS ver. 25. (Stats Guild, Chiba, Japan) was used for all statistical analyses. A *p* value of < 0.05 was considered to indicate statistical significance.

## Results

### Differences in the frequency and location of PACM according to H. pylori status

In the entire cohort of 6655 patients, 43 patients with a history of gastrectomy, 207 patients with specimens that were difficult for histological evaluation, and 475 patients whose *H. pylori* infection status could not be clearly determined were excluded, and the final analyses included 5930 patients (Additional file 1: Online Resource 1). There were 2039 patients (male/female ratio, 1145/894; mean age, 58.0 ± 14.7 years) in the CHI group, 3332 patients (male/female ratio, 1948/1384; mean age, 62.0 ± 12.7 years) in the PHE group, and 559 patients (male/female ratio, 272/287; mean age, 57.6 ± 17.6 years) in the NHI group (Additional file 1: Online Resource 1). The mean duration after eradication was 45.8 ± 52.5 months, ranging between 1 to 168 months, in the PHE group. PACM was observed in 10 of the 2039 patients (0.49%) in CHI group and in 25 of the 3332 patients (0.75%) in the PHE group, whereas PACM was not observed in the NHE group (Additional file 2: Online Resource 2).

Five- and two-point biopsies were performed in 582 and 5348 patients, respectively. The biopsy specimens from A1, IA, and B1 were collected in 582 patients, whereas the biopsy specimens from A2 and B2 were collected in 5930 patients. PACM was found in A2, A1, and B1 in 33, 1, and 1 patient, respectively, whereas PACM was not found in IA or B2 in the current study cohort (Additional file 3: Online Resource 3).

### Histopathology of PACM

**Fig. 1:**
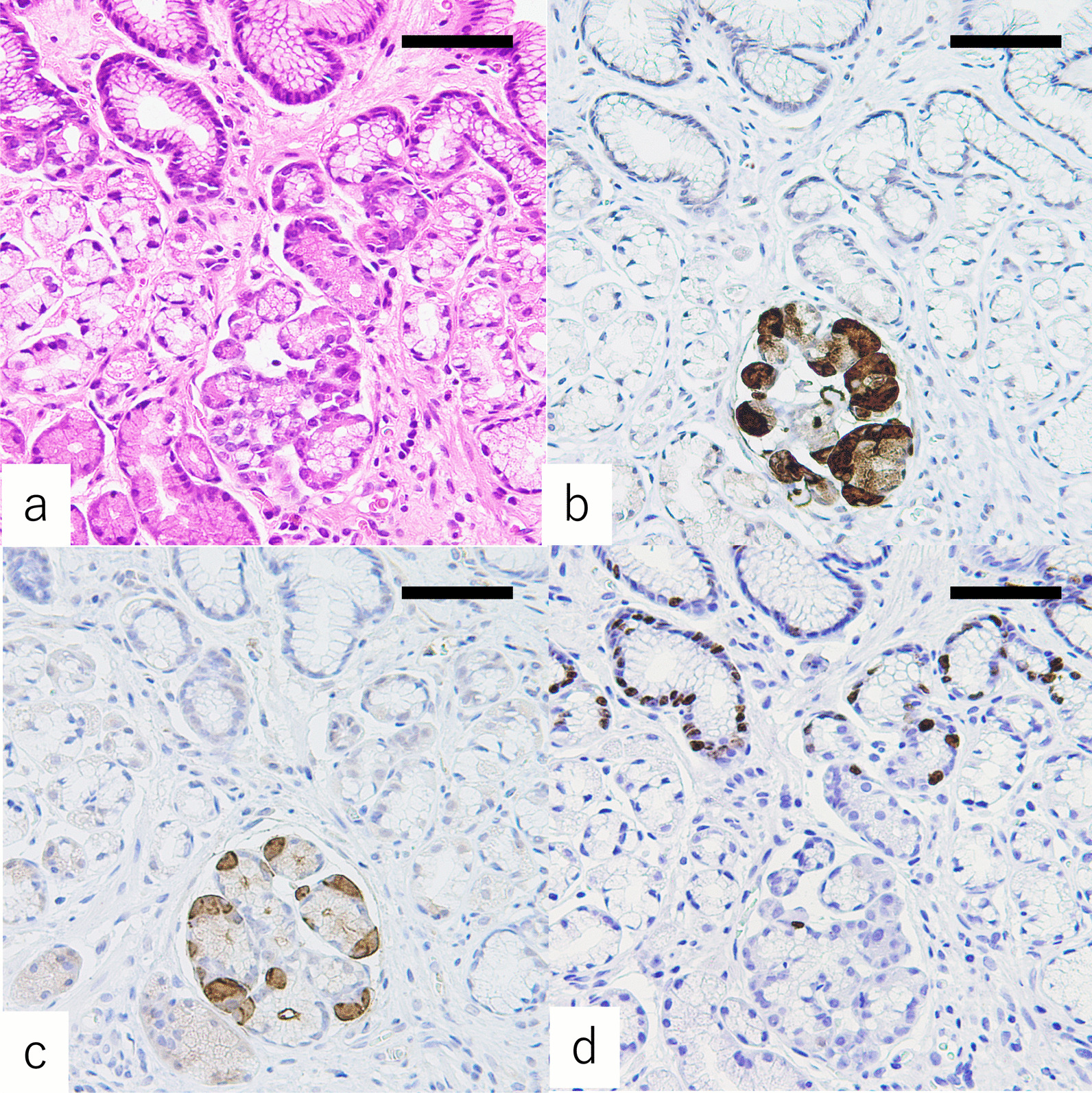
Immunostaining of pancreatic acinar cell metaplasia (PACM) lesions. Tissue specimens containing PACM obtained from the greater curvature of the antrum (A2) are stained with hematoxylin and eosin (**a**) and antibodies against Bcl-10 (**b**), α-amylase (**c**), and Ki-67 (**d**). Hematoxylin/eosin staining shows clusters of pancreatic acinar cells. Note that all pancreatic acinar cells are positive for Bcl-10 and that some pancreatic acinar cells are positive for α-amylase. Ki-67 expression is observed in the gastric neck near PACM. Scale bar: 200 μm

**Fig. 2:**
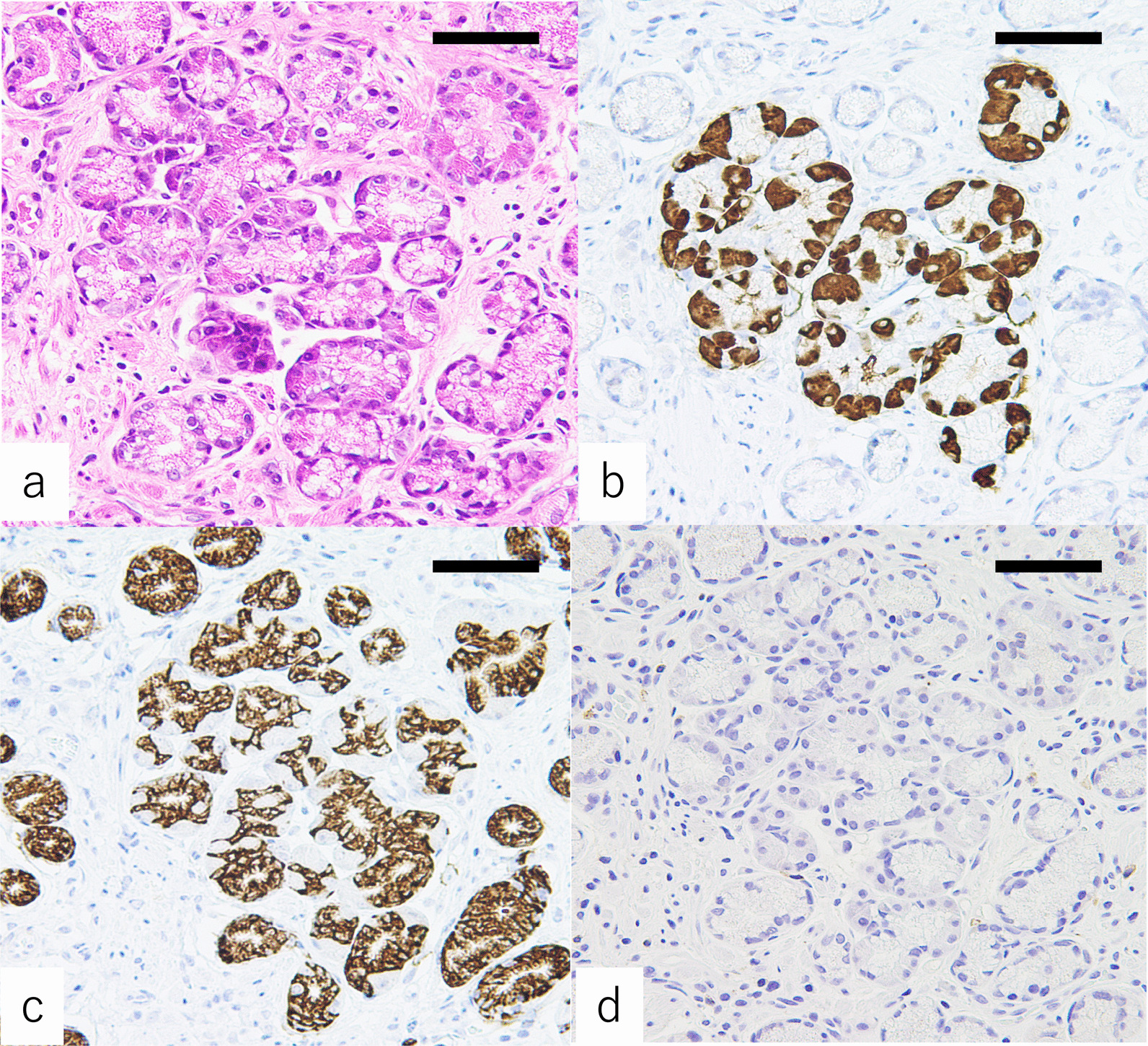
Immunostaining of PACM lesions. Tissue specimens containing PACM obtained from the greater curvature of the antrum (A2) are stained with hematoxylin and eosin (**a**) and antibodies against Bcl-10 (**b**), MUC6 (**c**), and pepsinogen-I (**d**). Hematoxylin/eosin staining shows clusters of pancreatic acinar cells. Note that all pancreatic acinar cells are positive for Bcl-10. All pyloric-type glands are positive for MUC6 but negative for pepsinogen-I. Scale bar: 200 μm

The areas of PACM comprised clusters of pancreatic acinar cell-like cells, exocrine cells with basophilic cytoplasm, and eosinophilic coarse granules (Figs. 1, 2). The cells of PACM were sporadically distributed from the neck to the bottom of the gastric mucosa but were never observed within the superficial and foveolar gastric epithelium. All PACM lesions recognized in the present study were accompanied by pyloric-type glands.

### Immunohistochemical staining

The exocrine cells with basophilic cytoplasm and eosinophilic coarse granules were positive for anti-Bcl-10 and anti-α-amylase antibodies. The staining by the anti-Bcl-10 antibody was stronger than that by the anti-α-amylase antibody in cells within the areas of PACM. Proliferating cells, which were positive for Ki-67, were more frequently observed in the neck near the areas of PACM but were uncommon in the small clusters of PACM (Fig. 1). All pyloric-type glands around the areas of PACM were positive for MUC6 but negative for pepsinogen-I (Fig. 2).

### Double immunofluorescence staining

**Fig. 3:**
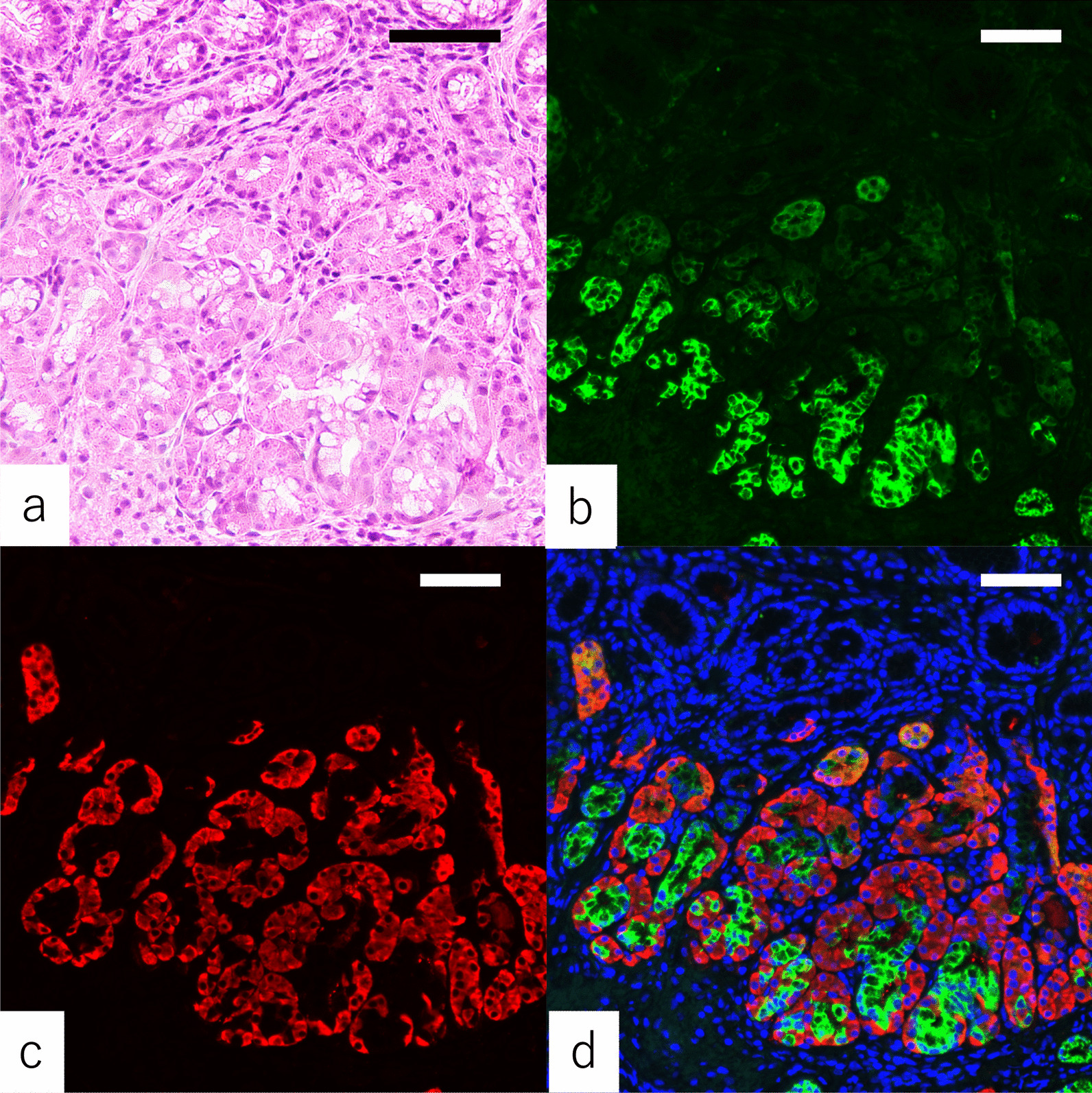
Double immunofluorescence staining for Bcl-10 and MUC6. Tissue specimens containing PACM obtained from the greater curvature of the antrum are stained with are stained with hematoxylin and eosin (**a**) and antibodies against MUC6 (red) (**b**) and Bcl-10 (green) (**c**). Merged images of specimens costained for Bcl-10 and MUC6 (**d**) are shown. Nuclei are counterstained with DAPI (blue). Although MUC6 and Bcl-10 did not overlap in many patients, there are some areas of overlap in the gastric neck. Scale bar: 200 μm

**Fig. 4:**
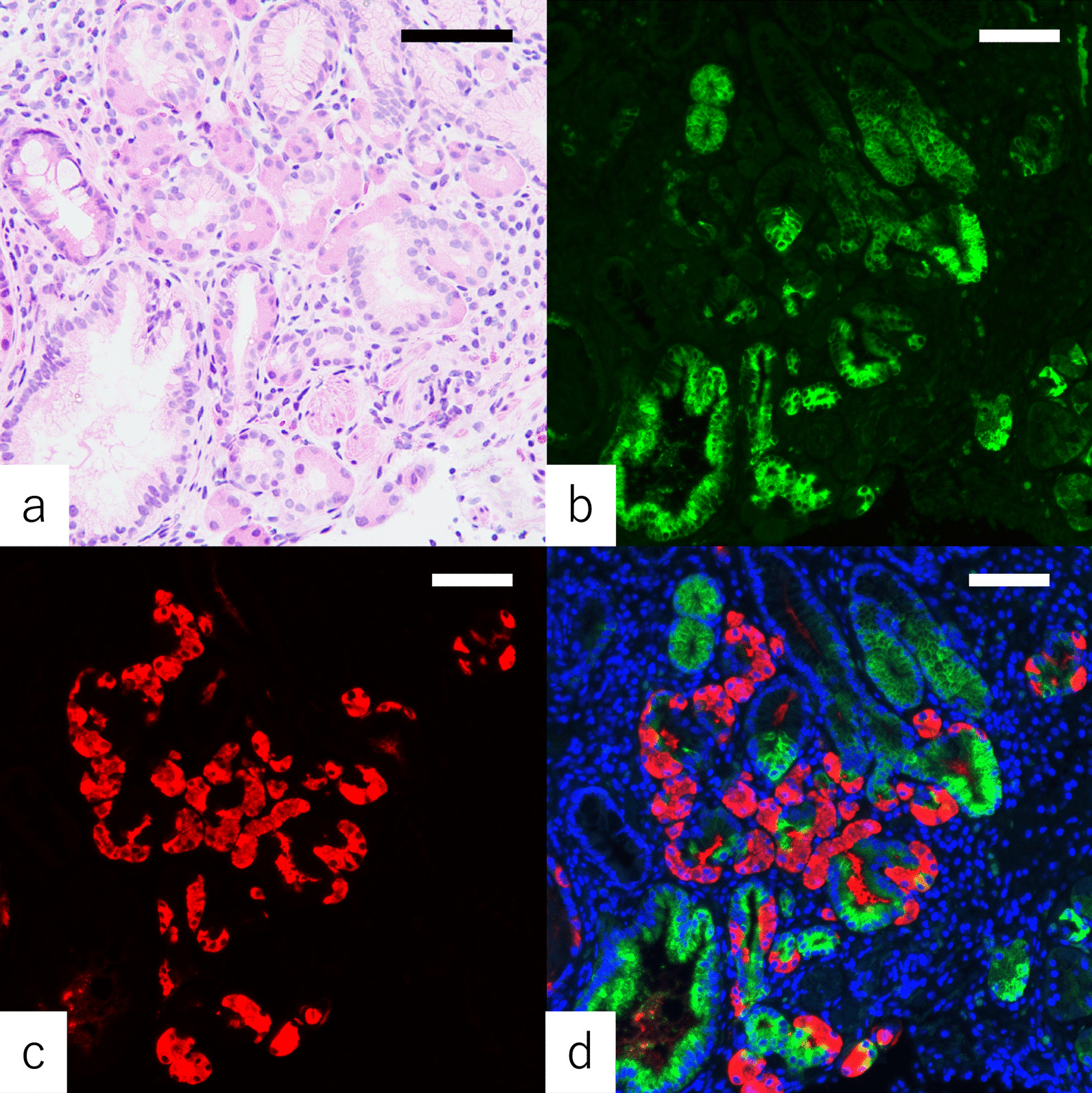
Double immunofluorescence staining for Bcl-10 and MUC6. Tissue specimens containing PACM obtained from the greater curvature of the middle body are stained with are stained with hematoxylin and eosin (**a**) and antibodies against MUC6 (red) (**b**) and Bcl-10 (green) (**c**). Merged images of specimens costained for Bcl-10 and MUC6 (**d**) are shown. Nuclei are counterstained with DAPI (blue). Scale bar: 200 μm

Double immunofluorescence staining showed that all cells in the areas of PACM were positive for Bcl-10 and that the pyloric-type glands around the areas of PACM were positive for MUC6 (Figs. 3, 4). Costaining for Bcl-10 and MUC6 was observed in the neck in some patients. All areas of PACM in A2 (Fig. 3) as well as in B2 were accompanied by pyloric-type glands (Fig. 4).

### The case–control study (A2, B2) of PACM in A2 and B2 with CHI

In the case–control study of patients with PACM in A2 and B2, we found that the inflammation scores in both A2 and B2 were lower in the PACM group than in the non-PACM group (*p* = 0.003 and *p* = 0.001, respectively) (Table 1). In contrast, no significant differences in activity, atrophy, or intestinal metaplasia scores were observed between the two groups. The atrophy scores were significantly lower in the PACM group compared with the non-PACM group (*p* = 0.02) (Table 1). Finally, albeit not statistically significant, the serum gastrin levels tended to be lower in the PACM group than in the non-PACM group (Table 1).

**Table 1:** Comparison of histopathological and clinical findings between PACM and no PACM groups of the case–control study

	PACM group (n = 10)	no PACM group (n = 30)	*P*-value*
*A2*			
Inflammation	2.3 ± 0.46	2.8 ± 0.48	0.003
Activity	1 ± 0.63	1.37 ± 0.71	0.14
Atrophy	1.1 ± 0.7	1.37 ± 0.91	0.48
IM	0.3 ± 0.64	0.77 ± 1.12	0.27
*B2*			
Inflammation	1.8 ± 0.6	2.6 ± 0.55	0.001
Activity	1 ± 0.89	1.13 ± 0.56	0.39
Atrophy	0.22 ± 0.42	0.5 ± 0.72	0.3
IM	0 ± 0	0.06 ± 0.36	0.56
Endoscopic atrophy	2.2 ± 1.25	3.6 ± 1.58	0.02
Serum gastrin (pg/mL)	145 ± 102.2	202 ± 112.4	0.08

Data are shown as mean scores ± standard error

PACM, pancreatic acinar cell metaplasia; IM, Intestinal metaplasia; USS, updated Sydney System; A2, the greater curvature of the antrum; B2, the greater curvature of the corpus

*P-value were calculated by the Mann–Whitney U-test

## Discussion

The biological significance and histogenesis of PACM in the gastric mucosa, which was first described in 1993 [1], remain uncertain [2]. Previous studies examining PACM in the stomach have been limited to small numbers of cases. Therefore, the present study with a large cohort might more closely reflect the true frequency of PACM in the stomach. In the present study, we examined 5930 specimens taken from patients with consent for research of *H. pylori* gastritis, while previous studies examined specimens biopsied in routine practices. Because we took biopsy specimens from five or two sites designated in USS for research of *H. pylori* gastritis, we evaluated the areas where PACM is found as precisely as possible. We found the frequency, location, and clinicopathological features of PACM in the context of *H. pylori* infection in the present study.

In the present study, PACM was present in patients with previous or current *H. pylori* infection but not in those without *H. pylori* infection, suggesting that *H. pylori* might play a crucial role in the development of PACM*.* Doglioni et al. reported that 75% of patients with PACM exhibited *H. pylori*-like organisms in the stomach only by histological examination [1], although the authors did not confirm the presence of *H. pylori* infection using other diagnostic methods. These organisms may be histological differential diagnoses as *Helicobacter heilmannii*, other organisms or mucous surface adherents. This study clearly shows that PACM is associated with *H. pylori* infection by clinically classifying CHI, PHE, and NHI . In addition, there was no significant difference in the proportion of patients with PACM between the two groups of CHI and PHE. In the previous study, atrophy, activity, and inflammation improve by *H. pylori* eradication, but intestinal metaplasia, pyloric metaplasia and pseudopyloric metaplasia are less likely to regress than atrophy, activity, and inflammation [6, 7]. In the present study, we found that PACM was also less likely to regress in the gastric mucosa. These results suggest that these gastric metaplasias remain to some extent after *H. pylori* eradication.

In the current cohort, most areas of PACM were in the antrum, in agreement with a previous study reporting that PACM was more frequent in the antrum (0.8%) than in the corpus (0.16%) [1]. In *H. pylori* gastritis, gastric inflammation and atrophy tend to be observed in the antrum rather than in the corpus [6], which might partially explain the higher frequency of PACM in the antrum than in the corpus among the patients with CHI. In addition, inflammation and atrophy scores were reported to be higher in the antrum even after a long eradication period in patients with *H. pylori* infection than in those without *H. pylori* infection [6]. PACM has been reported to occur near the glandular stomach–jejunal anastomosis in a rat model of duodenal content reflux, suggesting that gastric inflammation, atrophy, and bile acids might play an important role in the development of PACM in the antrum [5].

Two types of differentiation patterns are considered to underlie the pathogenesis of PACM: differentiation from the parietal cell lineage and differentiation from stem cells [4, 12]. Gastric stem cells are found in the proliferative zone of the neck [13], and the expression of Ki-67 observed in the neck in the present study provides support for the latter possibility. In addition, the overlap between Bcl-10 and MUC6 expression near the gastric neck by double immunofluorescence staining lends further support that PACM might be arising from this location. Furthermore, PACM was frequently accompanied by pyloric-type glands, implicating a relationship between PACM and pyloric-type glands. These findings indicate that pancreatic acinar cells develop from the proliferative zone of the neck without differentiating into pyloric-type glands. The mechanism of the differentiation into PACM remains uncertain. We could find the mechanism of the differentiation into PACM in the future by the analysis of the relationship between the pancreatic acinar cells and pyloric-type glands. In addition, heterotopic pancreas, a differential diagnosis of PACM, does not have pyloric-type glands. Heterotopic pancreas is found in the submucosa with pancreatic ducts and islets of Langerhans [1], whereas PACM is found in the lamina propria without them.

Among the metaplastic lesions found in the stomach, intestinal metaplasia is recognized as pre- and/or paracancerous atrophic lesions preceding gastric cancer [14]. Pyloric/pseudopyloric metaplasia has been reported in patients with CHI and advanced inflammation and atrophy [7]. In the present study, the inflammation scores in A2 were significantly lower in the PACM group than in the non-PACM group among those with CHI and the atrophy scores tended to be lower in the PACM group than in the non-PACM group among those with CHI. Additionally, the atrophy scores were significantly lower in the PACM group than in the non-PACM group. Krishnamurthy et al. reported that PACM was found in minimally inflamed gastric mucosa in pediatric gastric mucosa [4], suggesting that PACM might appear in mild inflammatory and atrophic conditions of the gastric mucosa. Severe inflammation and atrophy might alleviate PACM or might the emergence of other types of metaplasia from PACM. PACM was found in minimally inflamed gastric mucosa [4].

In the presence of *H. pylori* gastritis, the risk of gastric cancer has been reported to increase with the progression of atrophy [15, 16]. The present study findings do not clarify whether PACM might be associated with gastric cancer. However, there may be some relationship between PACM and carcinogenesis because the previous study reported that Barrett's adenocarcinoma and PACM frequently appeared at the esophagogastric junction when rats took PPI [17]. By investigating the mechanism of the differentiation of gastric mucosa into PACM, we could understand the pathogenesis of metaplasia when the gastric mucosa is damaged by *H. pylori* infection. Furthermore, we may find the mechanism of dysplasia and carcinogenesis in the future. Further studies are needed to examine the relationship between PACM and gastric carcinogenesis.

Other report showed that omeprazole was reported to cause PACM after six months of treatment in rats [18]. In humans, PACM is often observed in patients with autoimmune gastritis [3]. Hypergastrinemia is a shared feature between the oral administration of PPI and autoimmune gastritis; however, we did not find a relationship between hypergastrinemia and PACM in patients with *H. pylori* gastritis in the present study.

There are several limitations in the present study. First, this was a retrospective single-center study. Second, we examined biopsy specimens, which might not reflect the status of the entire gastric mucosa because PACM exhibits a patchy distribution pattern. Third, PACM may be associated with other diseases. We examined the biopsy specimens collected according to USS for the study of *H. pylori* gastritis, which were taken from sites without abnormity during the observation of esophagogastroduodenoscopy. Thus, we tried to exclude background factors other than *H. pylori* gastritis as possible, we may not exclude all diseases other than *H. pylori* gastritis completely.

## Conclusions

The following conclusion could be drawn by reviewing the many biopsy specimens collected daily for the research of *H. pylori* gastritis. We found that the development of PACM, especially in the antrum, was closely association with *H. pylori* infection.　Although PACM is uncommon, the study of PACM would be useful because there are a large number of patients with *H. pylori* gastritis. We may find the mechanism of carcinogenesis caused by *H. pylori* infection by elucidating epithelial morphological changes during the differentiation into PACM. Whether these changes in gastric mucosa influence due to *H. pylori* infection are associated with gastric cancer remains unclear, and future studies are necessary to elucidate the association of PACM with the development of gastric cancer.

## Supplementary Information

**Additional file 1: Online Resource 1.** Study flow chart. Among a total of 6655 eligible patients, 43 patients with a history of gastrectomy, 207 patients with specimens that were difficult for histological evaluation, and 475 patients whose H. pylori infection status could not be clearly determined were excluded. The final cohort of 5930 patients included 2039 patients with current H. pylori infection (CHI group), 3332 patients with confirmed H. pylori eradication (PHE group), and 559 patients without H. pylori infection (NHI group).

**Additional file 2: Online Resource 2**. Demographic details of the patients in each group. HP; Helicobacter pylori, HPE; Helicobacter pylori eradication, PACM; pancreatic acinar cell metaplasia All patients were classified into currentrly HP infection, after HPE and no HP infection groups. All patients in no HP infection group did not have PACM.Data are shown as mean scores±standard error.

**Additional file 3: Online Resource 3**. Proportion of patientets with PACM every five site biopsy HP; Helicobacter pylori, HPE; Helicobacter pylori eradication, PACM; pancreatic acinar cell metaplasia A1; the lessor curvature of the antrum, A2; the greater curvature of the antrum, IA; incisula angularis B1; the lessor curvature of the corpus, B2; the greater curvature of the corpus Data are shown as number of patients with PACM /number of total patients (percentage).

## Data Availability

Data and materials relevant to the study are included in this report. Further inquiries can be directed to the corresponding author.

## Abbreviations

PACM: Pancreatic acinar cell metaplasia
*H.** pylori*: *Helicobacter pylori*
PPI: Proton pump inhibitor
USS: Updated Sydney system
CHI: Current *H. pylori* infection
PHE: Post-*H. pylori* eradication
NHI: Non*-H. pylori* infection
A2: The greater curvature of the antrum
A1: The lesser curvature of the antrum
IA: The lesser curvature of the angulus
B1: The lesser curvature of the corpus
B2: The greater curvature of the corpus
