# Supplementary material for: Clinicopathological characteristics of pancreatic acinar cell metaplasia associated with Helicobacter pylori infection
Source: BMC Gastroenterol. 2022 Jun 7;22:289. doi: 10.1186/s12876-022-02338-2 (PMC9171985; doi:10.1186/s12876-022-02338-2)
Supplement: Supplementary file 1 — Additional file 1: Online Resource 1. Study flow chart. Among a total of 6655 eligible patients, 43 patients with a history of gastrectomy, 207 patients with specimens that were difficult for histological evaluation, and 475 patients whose H. pylori infection status could not be clearly determined were excluded. The final cohort of 5930 patients included 2039 patients with current H. pylori infection (CHI group), 3332 patients with confirmed H. pylori eradication (PHE group), and 559 patients without H. pylori infection (NHI group). [file 12876_2022_2338_MOESM1_ESM.pdf]

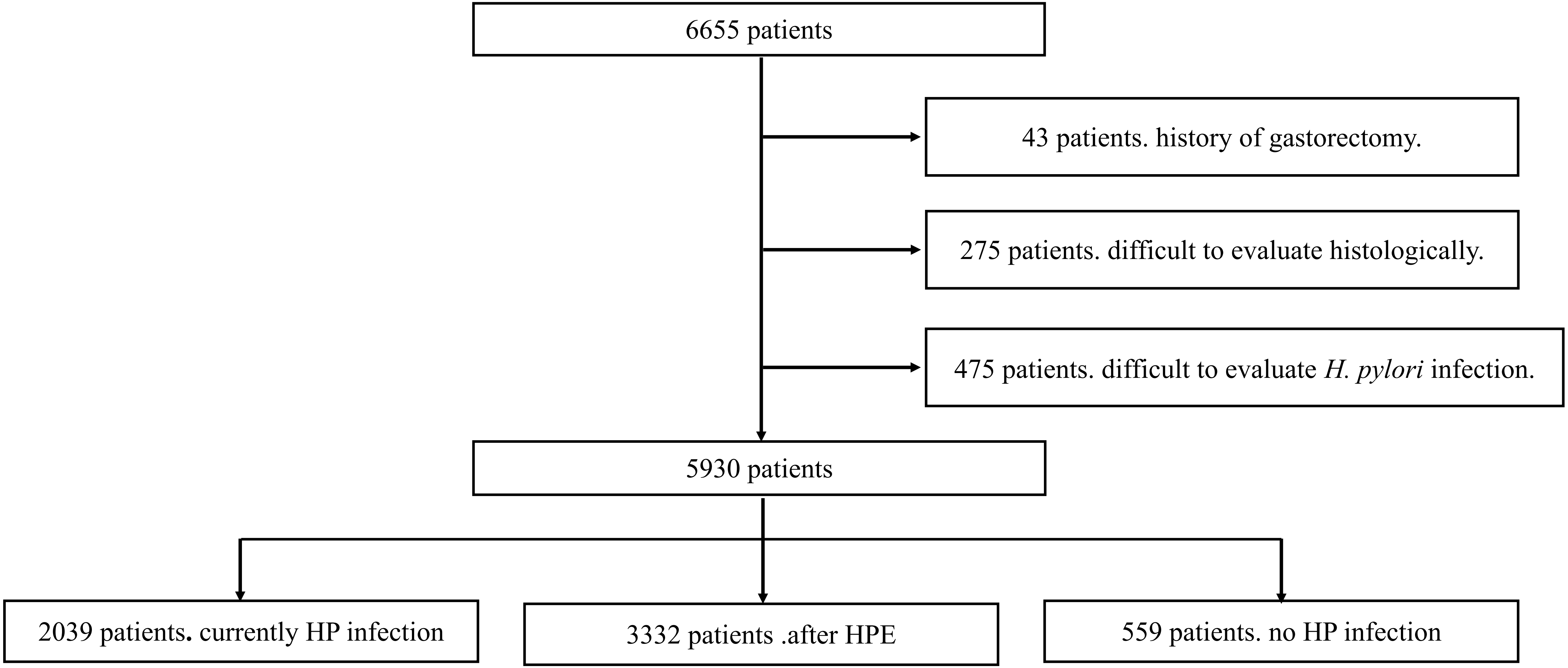
6655 patients
43 patients. history of gastorectomy.
275 patients. difficult to evaluate histologically.
475 patients. difficult to evaluate *H. pylori* infection.
5930 patients
2039 patients. currently HP infection
3332 patients .after HPE
559 patients. no HP infection
