# Supplementary material for: Clinicopathological characteristics of pancreatic acinar cell metaplasia associated with Helicobacter pylori infection
Source: BMC Gastroenterol. 2022 Jun 7;22:289. doi: 10.1186/s12876-022-02338-2 (PMC9171985; doi:10.1186/s12876-022-02338-2)
Supplement: Supplementary file 2 — Additional file 2: Online Resource 2. Demographic details of the patients in each group. HP; Helicobacter pylori, HPE; Helicobacter pylori eradication, PACM; pancreatic acinar cell metaplasia All patients were classified into currentrly HP infection, after HPE and no HP infection groups. All patients in no HP infection group did not have PACM.Data are shown as mean scores±standard error. [file 12876_2022_2338_MOESM2_ESM.pdf]

	currentrly HP infection (n=2039)		after HPE (n=3332)		no HP infection
	PACM		PACM		PACM
	+ (n=10)	- (n=2029)	+ (n=25)	- (n=3307)	- (n=559)
age	54.1±12.6	58.0±14.7	65.2±11.5	62.0±12.7	57.5±17.6
sex(male/female)	4/6	1141/888	8/17	1940/1367	272/287

Online Resource 2　Demographic details of the patients in each group
HP; Helicobacter pylori, HPE; Helicobacter pylori eradication, PACM; pancreatic acinar cell metaplasia
All patients were classified into currentrly HP infection, after HPE and no HP infection groups.
All patients in no HP infection group did not have PACM.
Data are shown as mean scores±standard error.
