# Supplementary material for: Clinicopathological characteristics of pancreatic acinar cell metaplasia associated with Helicobacter pylori infection
Source: BMC Gastroenterol. 2022 Jun 7;22:289. doi: 10.1186/s12876-022-02338-2 (PMC9171985; doi:10.1186/s12876-022-02338-2)
Supplement: Supplementary file 3 — Additional file 3: Online Resource 3. Proportion of patientets with PACM every five site biopsy HP; Helicobacter pylori, HPE; Helicobacter pylori eradication, PACM; pancreatic acinar cell metaplasia A1; the lessor curvature of the antrum, A2; the greater curvature of the antrum, IA; incisula angularis B1; the lessor curvature of the corpus, B2; the greater curvature of the corpus Data are shown as number of patients with PACM /number of total patients (percentage). [file 12876_2022_2338_MOESM3_ESM.pdf]

	A1	A2	IA	B1	B2
currently HP infection	1/91(1.1%)	9/2039(0.44%)	0/91(0%)	0/91(0%)	0/2039(0%)
after HPE	0/468(0%)	24/3332(0.72%)	0/468(0%)	1/468(0.21%)	0/3332(0%)
no HP infection	0/23(0%)	0/559(0%)	0/23(0%)	0/23(0%)	0/559(0%)
Total	1/582(0.17%)	33/5930(0.56%)	0/582(0%)	1/582(0.17%)	0/5930(0%)

Online Resource 3 Proportion of patientets with PACM every five site biopsy

HP; Helicobacter pylori, HPE; Helicobacter pylori eradication, PACM; pancreatic acinar cell metaplasia

A1; the lessor curvature of the antrum, A2; the greater curvature of the antrum, IA; incisula angularis

B1; the lessor curvature of the corpus, B2; the greater curvature of the corpus

Data are shown as number of patients with PACM /number of total patients (percentage).
